# Reverse shoulder arthroplasty for proximal humerus fracture using a dedicated stem: radiological outcomes at a minimum 2 years of follow-up—case series

**DOI:** 10.1186/s13018-015-0261-1

**Published:** 2015-08-22

**Authors:** Raffaele Garofalo, Brody Flanagin, Alessandro Castagna, Eddie Y Lo, Sumant G Krishnan

**Affiliations:** Shoulder Service, Upper Limb Unit, F. Miulli Hospital, Km 4 strada per Santeramo, Acquaviva delle fonti, Bari 70026 Italy; The Shoulder Center, Baylor University Medical Center, Dallas, TX USA; Shoulder and Elbow Unit, IRCCS Humanitas Institute, Milan, Italy; San Francisco Multi-Speciality Medical Group, San Francisco, CA USA

**Keywords:** Fracture, Proximal, Humerus, Reverse, Prosthesis

## Abstract

**Background:**

Complex proximal humeral fractures are very difficult to treat particularly in patients older than 65 years with an osteoporotic bone and tuberosities compromised. The goal of this paper is to evaluate radiological outcomes at mid-term follow-up of proximal humerus fractures treated with reverse shoulder arthroplasty using a dedicated fracture stem.

**Materials and methods:**

The study population included 98 patients who underwent reverse shoulder with a dedicated fracture stem for an acute proximal humerus fracture; 87/98 patients were available for analysis. There were 62 female and 25 male patients, and the mean age was 76.2 years at the time of surgery (range 61–90 years). Clinical and radiological outcomes were evaluated at a mean follow-up of 27 months after surgery.

**Results:**

Average active elevation was 137.7°, external rotation 29.1°, and internal rotation 40.7°. Overall, the tuberosity healing rate was 75 %. There was a significant increase in active anterior elevation, external rotation, and internal rotation among patients who demonstrated radiographic evidence of tuberosity healing. All tuberosity nonunions (21 cases) occurred preferentially in females, but this number did not reach statistical significance.

**Conclusion:**

RSP using a dedicated stem is a very viable solution to treat complex humerus proximal fracture. Reliable restoration of elevation can be expected. However, in patients in whom tuberosity healing occurs, a better active elevation other than restoration of active rotational movement can be observed.

## Introduction

The majority of proximal humerus fractures in patients over the age of 65 are minimally displaced and can be treated nonoperatively with satisfactory clinical outcomes [1]. However, certain fractures in this age population requiring surgical treatment are often not amenable to repair because of poor bone quality, potential loss of fixation, and a high risk of nonunion or osteonecrosis. In these cases, primary arthroplasty is a viable option. Hemiarthroplasty (HA) has historically been considered the standard of care for patients greater than 65 years of age in whom arthroplasty is performed for proximal humerus fracture. Clinical studies have demonstrated a significative advantage in terms of pain and quality of life after HA compared with nonoperative treatment for displaced proximal humerus fractures in the elderly [2]. However, HA for fracture remains a challenging procedure as clinical outcomes are largely influenced by proper implant placement and tuberosity healing which has yielded unpredictable results with respect to functional outcomes [3–7].

Recently, reverse shoulder arthroplasty (RSA) has emerged as an alternative option for the treatment of acute, comminuted proximal humeral fractures in elderly patients [8–10]. RSA is an attractive option in this population because the design does not rely on a functioning rotator cuff for overhead shoulder range of motion. Furthermore, patients with RSA typically require less intensive and prolonged physical therapy to regain functional shoulder range of motion [11]. The purpose of this study was to evaluate mid-term clinical and radiographic outcomes in a cohort of elderly patients treated with RSA using a dedicated fracture stem for an acute proximal humerus fracture. Our hypothesis was that RSA with a dedicated fracture stem leads to satisfactory clinical outcomes that can be correlated to the presence of radiographic healing of the greater tuberosity.

## Materials and methods

**Fig. 1:**
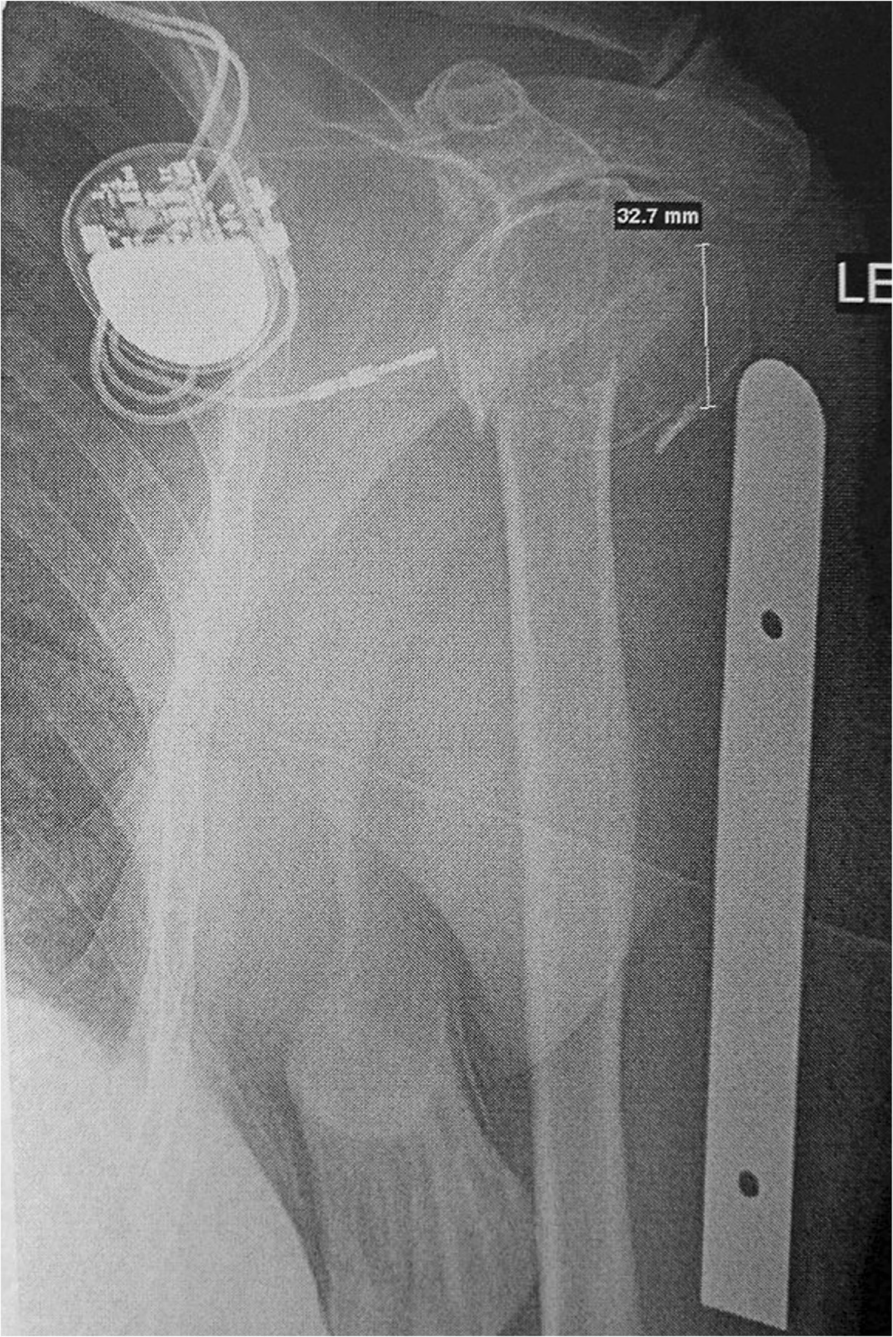
Preoperative templating measuring the greater tuberosity height is shown. In this case, it was 32.7 mm. A metallic scaled ruler of 10-cm length is used to calculate radiographic magnification and obtain actual number

From January 2009 and March 2012, 98 patients underwent RSA for the treatment of an acute proximal humerus fracture using a specific dedicated stem by two surgeons at two different institutions (SGK and RG). All patients had acute fractures and underwent the operative treatment in a period between 3 and 15 days after trauma. Sixteen patients were lost at final follow-up, leaving 87 patients for final analysis. There were 62 female and 25 male patients, and the mean age was 76.2 years at the time of surgery (range 61–90 years). At preoperative time, patients underwent X-ray and CT scan evaluation. CT scan investigation was useful for a better evaluation and classification of fracture. Bilateral full-length X-rays were performed to template the approximate height of the prosthesis for insertion. The measurement of height of the greater tuberosity fragment was carried out for the same goal (Fig. 1). All the patients had a three- or four-part proximal humerus fracture or a two-part fracture with a split of the humeral head. Clinical and radiographic follow-up was performed on all 87 patients at an average of 27 months (range 24–32 months) postoperatively. The primary outcome was postoperative range of motion in active elevation, external rotation, and internal rotation. Two physicians not involved in patient care take the measurement of shoulder range of motion (ROM) using a goniometer. The presence of pain was evaluated using a Visual Analog Scale (VAS) from 0 to 10, where 0 is pain free and 10 maximum pain. The secondary outcome was the radiographic presence of tuberosity healing. Standardized radiographs utilizing a true anteroposterior view in internal and external rotation, an axillary lateral view, and a scapular Y view made at final follow-up visit were used to judge tuberosity healing. One very experienced musculoskeletal radiologist evaluated all radiographic films.

Radiographs were also used to judge implant loosening on the humeral or glenoid side as well as scapular notching. Humeral component loosening was measured using the grading system described by Sperling [12]. Glenosphere and baseplate fixation was graded in a manner previously described as stable, at risk, or loose [10]. Scapular notching was measured using the grading system of Sirveaux et al. [13].

Statistical analysis was performed using SPSS (Statistical Package for the Social Sciences) software. Continuous data were evaluated using two-tailed unpaired *t* tests to compare the equality of variance. Categorical data was analyzed using chi-square and Fisher’s exact tests. Regression analysis was performed to assess correlation for Pearson’s correlation coefficient. Statistical significance was indicated at *p* < 0.05. Active shoulder ROM was correlated with the presence of radiographic healing of the greater tuberosity.

This retrospective study was approved by the Institutional Ethics Committee of Miulli Hospital and was conducted in accordance with the latest version of the Helsinki Declaration. All patients were informed about the study and signed an informed consent form.

### Surgical technique

**Fig. 2:**
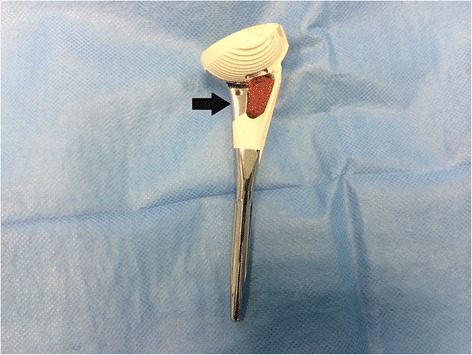
The fracture-specific stem used in this series is demonstrated. Autologous humeral head cancellous bone is harvested and inserted in the metaphyseal window prior to implantation (*arrow*)

All surgeries were performed in a modified beach chair position with the head of patient elevated between 20° and 30° and the ipsilateral scapula supported. An Aequalis™ Reversed Fracture (Tornier, Edina, MN) prosthesis shoulder was implanted in all cases. A deltopectoral approach was used. The biceps tendon was tenodesed to the upper border of the pectoralis major in all cases. After removal of the humeral head fragment, four no. 5 nonabsorbable sutures were placed through the infraspinatus and teres minor at the bone-tendon junction to control the greater tuberosity fragment. The lesser tuberosity and attached subscapularis are identified and tagged for later repair around the prosthesis. The baseplate and glenosphere were then implanted in a standard fashion. We prefer to use a 4-mm lateralized glenosphere in most cases in order to reduce the risk of scapular notching. The humeral shaft is exposed and prepared with hand reamers until there is gentle cortical resistance. A humeral trial is then placed. Appropriate height of the humeral implant and length of the greater tuberosity fracture fragment were determined preoperatively based on a previously described technique [14]. This measurement was confirmed intraoperatively. If any discrepancy existed between the pre- and intraoperative measurements of tuberosity length, the intraoperative measurement was always utilized. Humeral version was then set by placing the arm in a neutral position at the side and pointing the humeral component/tray toward the glenosphere. Two drill holes were then placed on either side of the bicipital groove and 3-mm cottony Dacron sutures were placed through each and passed through the rotator cuff for vertical tension-band fixation of the tuberosities. The humeral component was cemented distally in all cases for hybrid fixation. Once the cement cured with the humeral component seated to it is at appropriate height and version, a trial reduction was performed with a constrained polyethylene trial onto the humeral component to confirm satisfactory positioning and stability without evidence of impingement during range of motion. The humeral component is then dislocated, and the final constrained polyethylene liner is impacted into place. The four medial suture limbs previously passed through the infraspinatus and teres are then cerclaged around the neck of the prosthesis before performing a final reduction. Humeral head allograft is then harvested and placed into the metaphyseal window of the prosthesis in order to aid in tuberosity healing and implant stability (Fig. 2). The greater tuberosity was reduced and gently held in its anatomic position with a small 2-mm nonpenetrating awl. Two of the four previous suture limbs cerclaged around the neck of the prosthesis were tied; the two remaining sutures were placed through the subscapularis at the bone-tendon junction and tied with the lesser tuberosity held in an anatomic position with the pointed awl. The two 3-mm cottony Dacron are then passed through the rotator cuff (one anterior to posterior and the other from posterior to anterior), thereby creating vertical tension-band fixation of the tuberosities to the shaft. After surgery, the ipsilateral extremity was placed into a simple Velpeau arm sling with the arm resting at the side prior to extubation.

### Postoperative care

Postoperatively, patients are placed in a sling for 5 weeks. Immediate passive motion is started with flexion to 90° and external rotation to 30°. Full passive forward flexion begins at week 5. Active assisted motion in all planes is initiated starting at week 7. At this time, the use of arm for light home activities was allowed.

## Results

**Fig. 3:**
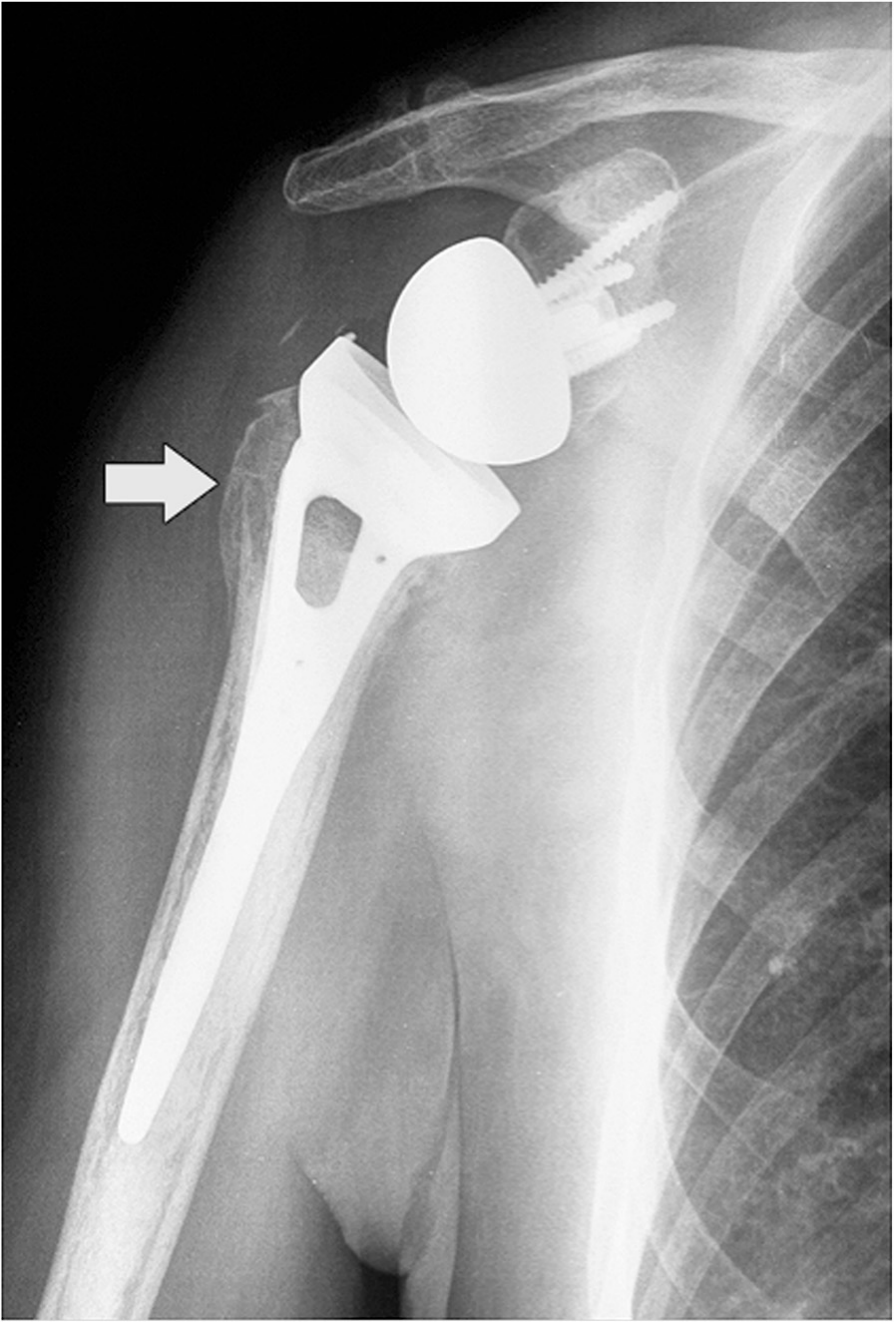
AP radiograph at 28 months of follow-up in a 74-year-old woman showing a healed greater tuberosity (*arrow*)

Most parts of the patients were pain free at rest and during activities. Only five patients referred an occasional pain (VAS 3–4) during prolonged arm activities. At final follow-up, average active elevation was 137.7° (range 97°–155°), external rotation 29.1° (range 55°–9°), and internal rotation 40.7° (20°–50°). The overall radiographic tuberosity healing rate was 75 % (Fig. 3).

**Fig. 4:**
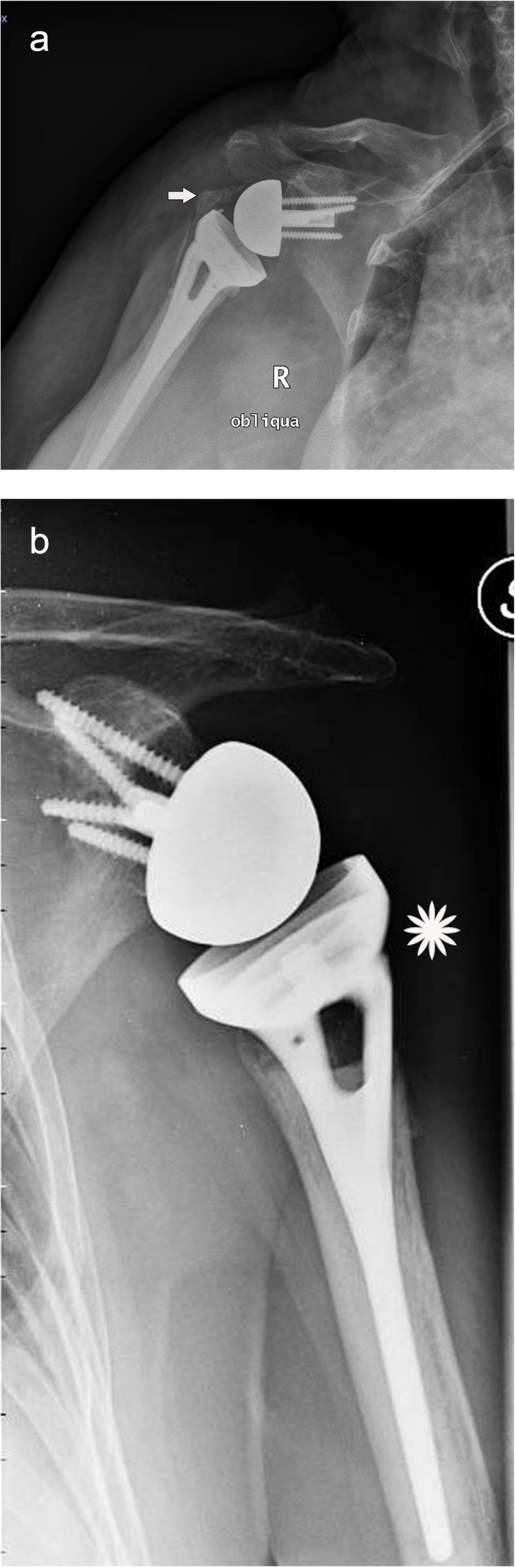
**a** AP radiograph at 30 months postoperatively in an 81-year-old woman showing a partial union of the greater tuberosity and a partial superior migration. The *white arrow* indicates the part of the greater tuberosity that migrates superiorly with respect to the metaphyseal component of the prosthesis. The R oblique indicates AP oblique view of a right shoulder. **b** AP radiograph at 28 months postoperatively in a 72-year-old woman showing complete resorption of the greater tuberosity. In fact, there is no more bone lateral to the metaphyseal part of the stem (*asterisk*)

If the tuberosity was healed, the patients had average active elevation of 145.3° (range 137°–155°), external rotation of 34.3° (25°–55°), and internal rotation of 45.6° (40°–50°). Among patients without a healed greater tuberosity, average active elevation was 114.1° (range 97°–124°) (*p* < 0.0001), external rotation 12.9° (range 9°–16°) (*p* < 0.0001), and internal rotation 25.7° (range 20°–39°) (*p* < 0.001) (Fig. 4).

The postoperative active elevation (*p* = 0.1), external rotation (*p* = 0.11), and internal rotation (*p* = 0.39) did not correlate with patient age. There were also no detectable differences in active elevation (*p* = 0.11), external rotation (*p* = 0.4), or internal rotation (*p* = 0.5) with duration of postoperative follow-up. Similarly, we did not identify any detectable differences in postoperative motion with respect to gender (Table 1).

**Table 1:** Clinical outcome about the range of motion data at final follow-up

		Range of motion					
		Anterior elevation	*p* value	ER	*p* value	IR	*p* value
Gender	Male	135.2 ± 31.5	0.78	33.5 ± 15.6	0.3	39 ± 17.3	0.78
	Female	138.2 ± 20.9		28.1 ± 14.7		41.1 ± 21.5	
Tuberosity healing	Healed	145.3 ± 19.3	<0.0001	34.3 ± 11.8	<0.0001	45.6 ± 18.9	<0.001
	Not healed	114.1 ± 15.8		12.9 ± 11.6		25.7 ± 19.1	

In this table, the gender and tuberosity healing independently are related to the final range of motion

Using tuberosity healing as an independent outcome variable, we found that the average age of patients who had a healed greater tuberosity was 75.6 whereas the average age of patients with a tuberosity nonunion was 78.1 (*p* = 0.29) (Table 2). All tuberosity nonunions or resorption occurred in females (21 cases), but this number did not reach statistical significance (*p* = 0.1). Tuberosity healing was influenced by the fragmentation of the greater tuberosity observed during surgery. Very comminuted fractures are associated with resorption.

**Table 2:** Comparison of tuberosity healing with gender and age

Tuberosity healing	Healed	Not healed	*p* value
Age (years)	75.6 ± 7	78.1 ± 8.6	0.29
Male	25	0	0.10
Female	41	21	

**Fig. 5:**
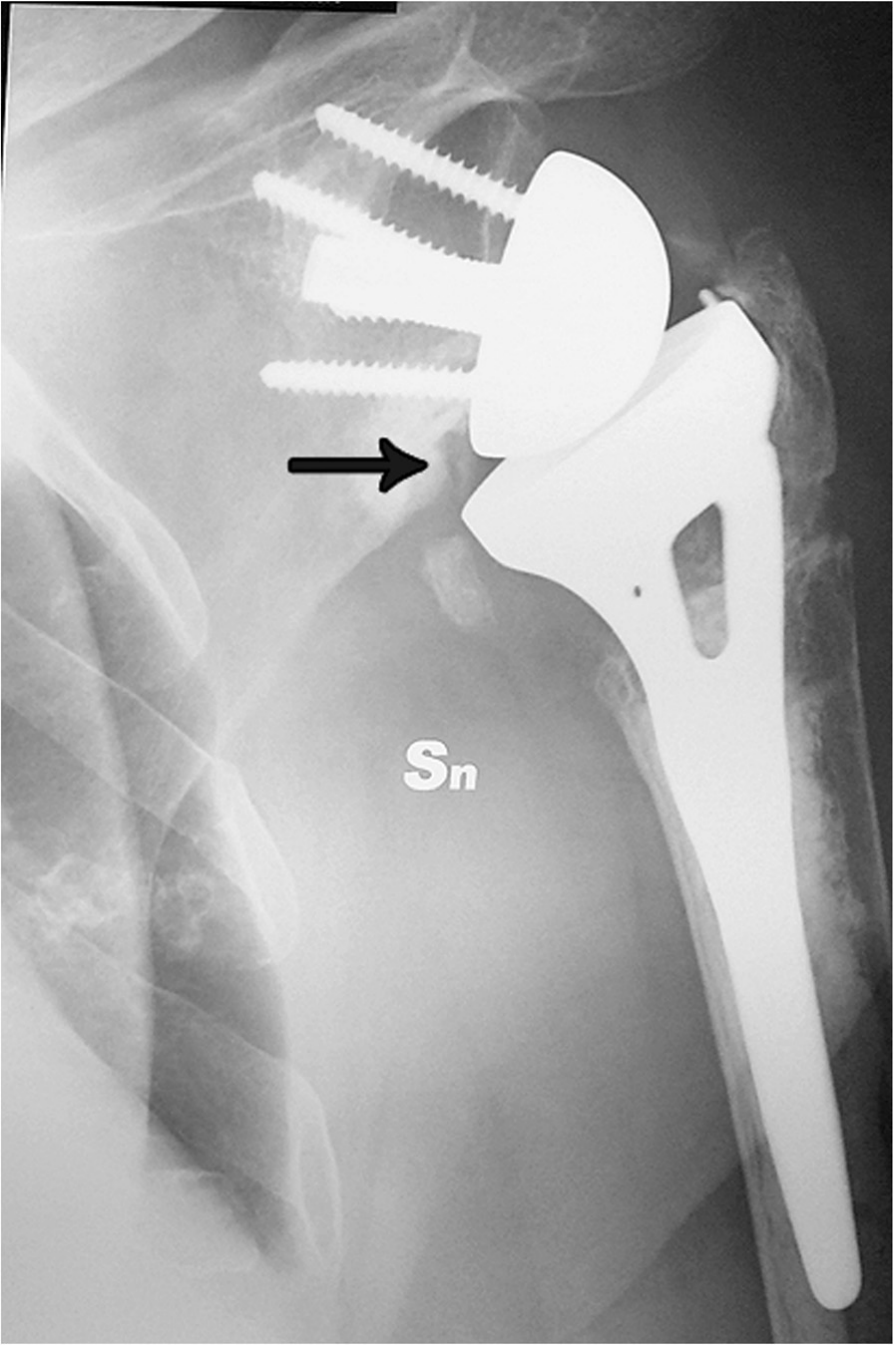
AP radiograph of a 76-year-old woman at 24 months postoperatively demonstrating grade 1 scapular notching (*black arrow*). Sn means left

One patient had a superficial infection that was treated successfully with oral antibiotics. Two patients had a late deep infection (>1 year after surgery) and were treated with prosthetic removal and an antibiotic spacer. In one case, a transient neuropraxia of the radial nerve of the ipsilateral arm was observed, which resolved at 8 months postoperatively. Scapular notching was observed in only case (Fig. 5). This was the only patient in whom a standard and not a 4-mm lateralized glenosphere was implanted. No loosening of the humeral or glenoid components and no episodes of dislocation/instability were observed in this series.

## Discussion

Surgical management for displaced three- and four-part proximal humeral fractures in the elderly remains a challenge. Despite the advent of locking plate technology, open reduction and internal fixation (ORIF) of complex proximal humeral fractures in this group of patients is often not a viable option because of high complication rates [15].

For many years, HA has been considered the standard for the treatment of complex, displaced proximal humeral fractures in the elderly. However, HA carries its own set of technical challenges including proper prosthetic height, version, and tuberosity fixation. These are all critical factors to ensure a satisfactory functional outcome, and dedicated fracture stems have been previously shown to improve radiographic tuberosity healing rates and functional outcomes [3, 16].

RSA has become an attractive option for treating displaced proximal humerus fractures in the elderly because it relies primarily on deltoid muscle function and may minimize the need for anatomic tuberosity healing/rotator cuff function. Several studies have demonstrated more predictable functional outcome after RSA compared with HA. Gallinet et al. previously compared the outcomes of 17 patients treated with HA and 16 patients treated with RSA after a proximal humeral fracture. Patients who underwent RSA had significantly better abduction, elevation, and constant scores at final follow-up [17]. Garrigues et al. performed a retrospective comparison of 12 patients with HA and 11 patients with RSA for the treatment of an acute proximal humeral fracture. Their series demonstrated significantly better elevation and functional outcomes in patients with RSA [18]. Lenarz et al. retrospectively reviewed 30 patients with a mean age of 77 years who had undergone a primary RSA for the treatment of a three- or four-part proximal humeral fracture. They reported satisfactory clinical outcome scores and pain relief at a minimum follow-up of 12 months. Their series demonstrated a 10 % complication rate; however, no complication required another operation [19]. Sirveaux et al. reported that HA for fracture resulted in a very wide range of anterior elevation (between 10° and 180°), whereas in cases of RSA, the results were clustered around 110°, never greater than 150°. Furthermore, they demonstrated that anatomic healing of the tuberosities provides improved active internal and external rotation [20]. Cazeuneve et al. also noted that recovery of active external rotation was better in cases where the tuberosities had been fixed [9]. A recent paper comparing results of HA versus RSA for displaced fractures of the proximal humerus in elderly patients showed that clinical outcomes of patients who were treated with RSA had more significantly better and more consistent results irrespective of tuberosity healing [21]. Table 3 summarizes the main outcome reported by several previous studies.

**Table 3:** The main findings of previous and the present study are reported

Study	Active forward elevation (mean degree)	Active external rotation (mean degree)	Percentage of GT healing
Gallinet et al. [17]	97.5	9	NA
Garrigues et al. [18]	122	33	NA
Lenarz et al. [19]	139	27	NA
Sirveaux et al. [20]	107	10	NA
Cazeneuve et al. [9]	NA	NA	NA
Bufquin et al. [8]	97	8 neutral 30 abduction	NA
Klein et al. [22]	122	25	NA
Cuff et al. [21]	139	24	83 %
Our series	145 (GT healed)	34.3 (GT healed)	75 %
	114 (GT not healed)	12.9 (GT not healed)	

*NA* not reported, *GT* greater tuberosity

In our series, performing RSA with a dedicated fracture stem for the treatment of acute proximal humerus fractures resulted in a radiographic tuberosity healing rate of 75 %. Contrary to previous reports, we found that tuberosity healing is associated with significantly better active forward flexion, internal rotation, and external rotation. Patients in which the tuberosity was not healed still demonstrated satisfactory range of motion that is similar to previous reports in the literature. It is interesting to note that all cases of tuberosity nonunion/resorption were observed in female patients only which may be a result of poorer bone quality with less capacity for healing.

This study does have several weaknesses. We performed a retrospective evaluation of patients with no control group. Patients included were operated on by two different surgeons which may predispose to subtle differences in the execution of critical components of the procedure that otherwise may have affected the final outcome.

In summary, RSA using a dedicated fracture stem results in satisfactory range of motion at mid-term follow-up in the surgical treatment of displaced proximal humerus fractures in elderly patients. Radiographic tuberosity healing appears to result in improved active range of motion in all planes.
